# Starting HMV at home: a reasonable option for many patients?

**DOI:** 10.1186/s12890-022-02195-5

**Published:** 2022-11-09

**Authors:** Ries J.M. van den Biggelaar, A. Hazenberg, N. A.M. Cobben, M. A. Gaytant, D. A.M.P.J. Gommers, P. J. Wijkstra

**Affiliations:** 1 Department of Pulmonary Diseases, Department of Intensive Care Medicine and Home Mechanical Ventilation, Erasmus Medical Centre Rotterdam, Rotterdam, The Netherlands; 2 Department of Pulmonary Diseases/Home Mechanical Ventilation, University of Groningen, University Medical Centre Groningen, Groningen, the Netherlands; 3 Groningen Research Institute for Asthma and COPD (GRIAC), University of Groningen, University Medical Centre Groningen, Groningen, the Netherlands; 4 Department of Pulmonary Diseases/Home Mechanical Ventilation, University Medical Centre, Maastricht, the Netherlands; 5 Department of Pulmonary Diseases/Home Mechanical Ventilation, University Medical Centre, Utrecht, the Netherlands; 6 Department of Home Mechanical Ventilation, Erasmus Medical Center Rotterdam, Doctor Molewaterplein 40, 3015 GD Rotterdam, the Netherlands

**Keywords:** Home mechanical ventilation, Telemonitoring, Patient selection, Health care organization

## Abstract

**Background and objective:**

In the current study, we undertook a more detailed exploration of the reasons why patients undergoing HMV were screened out of a recently published study in order to better understand how applicable home initiation of HMV is under real life conditions.

**Methods:**

All referred patients who had an indication for starting HMV were screened to participate in the Homerun study. In this trial 512 patients were screened out of the study. Those patients not enrolled in the trial were divided into the following 3 groups: (1) those not fulfilling the inclusion criteria; 2) those meeting the exclusion criteria and 3) those excluded on the basis of medical or organisation reasons. Each group was then further divided into those who would likely have been suitable for initiation of HMV at home in real world practice and those who were unsuitable.

**Results:**

Based on inclusion criteria (group 1) 116 patients could not start HMV in real life, while this was 245 patients in the study. Based on the exclusion criteria (group 2) 11 patients could not start in real life while this was 79 in the study. One hundred and eighty-eight could not be enrolled in the study due to medical and organisational reasons ( group 3), while in real life this was only 95.

**Conclusion:**

This study indicates that more than 55% of patients who did not participate in the Homerun study could have started HMV at home in real life.

## Introduction

Home mechanical ventilation (HMV) is an effective treatment for patients with chronic hypercapnic respiratory failure, resulting in improved clinical symptoms, health status and survival in patients with both restrictive and obstructive pulmonary disorders [1]. Consequently, we currently see that rapid growth in the number of patients on HMV is occurring [1]. In the Netherlands, it is common to initiate HMV in an inpatient setting with monitoring of gas exchange; however, the observed increase in demand for HMV represents an extra burden to the hospitals with regard to the availability of hospital beds. In addition, hospitalisation is stressful for patients as they have to leave their social environment.

More importantly, the existing patient tailored care system at home in every individual patient, often a combined team of professional and informal caregivers, will not be positively affected by hospitalisation. Therefore, there is a need for alternative ways of initiating HMV. With this in mind, we designed a national study, The Dutch Homerun trial, [2] based on previously designed local studies on home initiation of HMV, [3, 4] in which we compared initiation of HMV at home with inpatient initiation. We showed that home initiation was equally effective as hospital initiation. However, a large group of patients did not meet the inclusion criteria (84%), therefore, they did not get the opportunity to start ventilatory support at home.

Therefore it is important to know the number of patients that could be candidate for initiate HMV at home. So we need to know if subjects were solely excluded due the study design and strict enrolment criteria or as a consequence of truly being unsuitable for home initiation of NIV based on clinical or social factors. The findings could have a significant impact on HMV protocols for initiating HMV in the home setting with implications for resource and personnel allocation in the future. Therefore, we undertook this study to identify and review the reasons why patients were excluded from the Homerun study to better understand the number of individuals in usual clinical practice who may be eligible for initiation of NIV in the home setting.

## Methods

During the inclusion period of the Homerun study, all patients who qualified for HMV were screened. For participation, they had to meet the following inclusion criteria: diagnosis of neuromuscular disease (NMD) or thoracic cage disorder with the indication for HMV based on complaints of alveolar hypoventilation (fatigue, headache of dyspnoea) combined with all following criteria: [2].

- Arterial carbon dioxide > 6.0 kPa daytime, or arterial or transcutaneous carbon dioxide > 6.0 kPa at night, or orthopnea as a result of diaphragm paralysis.
- Over 18 years of age.
- In the opinion of the supervising centre for HMV, the patient had a sufficient social or professional network to permit the safe initiation of HMV in the home.

The following exclusion criteria were applied:

- Previously received CPAP therapy or non-invasive ventilatory support.
- Requiring invasive ventilatory support.
- Living in a nursing home.

**Fig. 1:**
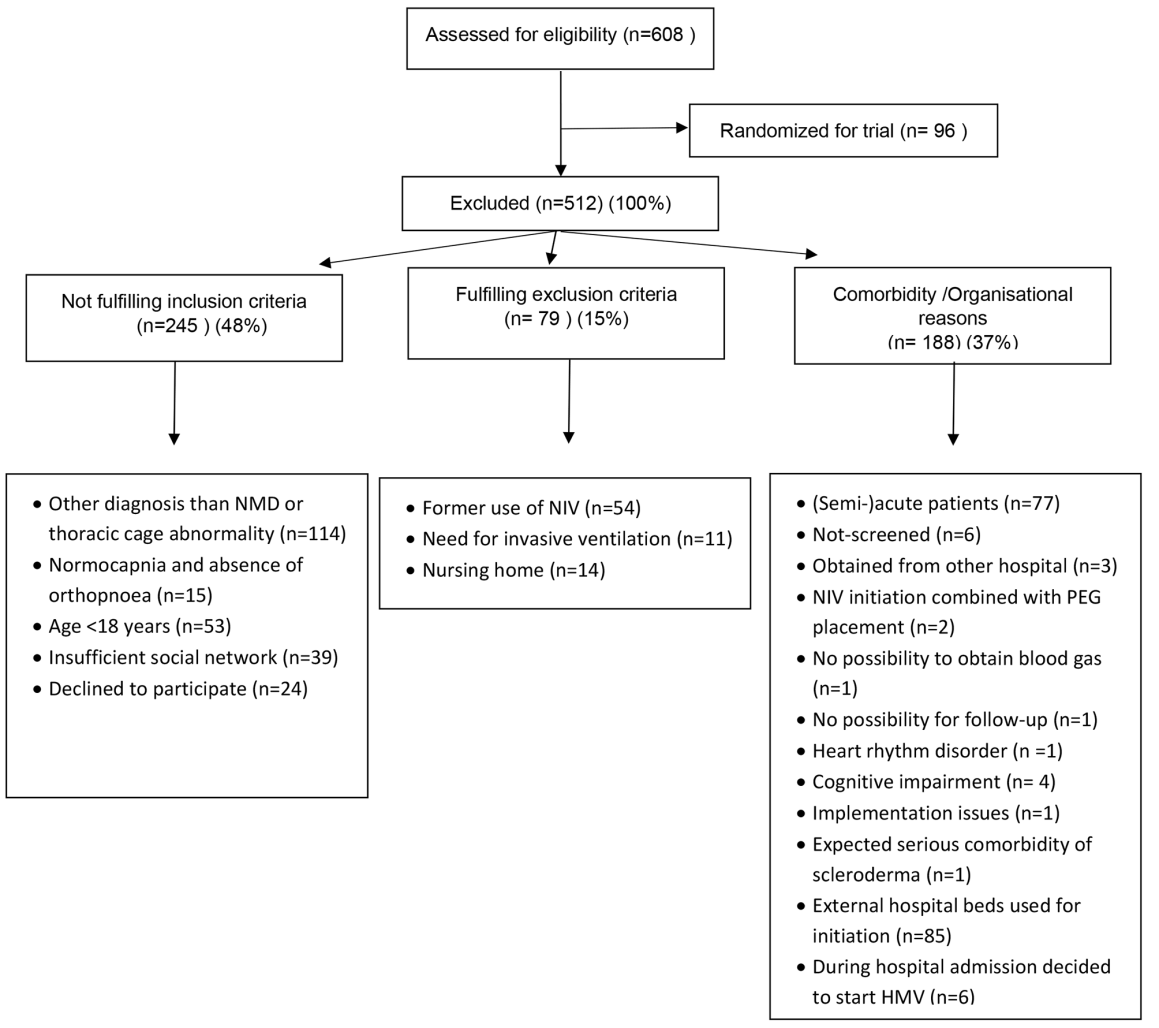
Flowchart of patient exclusion divided into main groups and subcategories

We categorised all non-eligible participants into 3 main groups by the following order: (i) those who did not meet the inclusion criteria (ii) those who met an exclusion criterion and (iii) those excluded from the study based on comorbidities or organisational reasons. The main groups were further subcategorized (see Fig. 1). The reason for not participating was evaluated to determine if the exclusion was solely based on the Homerun study design or whether there was a decisive factor that prevented the save initiation of HMV at home.

Each of these 3 groups were then further divided into those in whom it was felt exclusion from the study was solely based on the Homerun study design, and those in whom there was another reason for preclusion from the ability to initiate HMV. We use the term real life for the patient situation out of the study designed factors for not participating and into barriers which prevent from initiation at home in a daily practice work situation.

## Results

During the recruitment period of the Homerun study, a total of 608 patients were screened and commenced on HMV. However, only 96 participants were eligible for the study, leaving 512 patients to be set up on HMV outside of the study. The reasons for not participating were categorized into three main groups (as mentioned in the Methods section). These groups were further subdivided to gain detailed insight (see Fig. 1).

### Patients not fulfilling the inclusion criteria

**Fig. 2:**
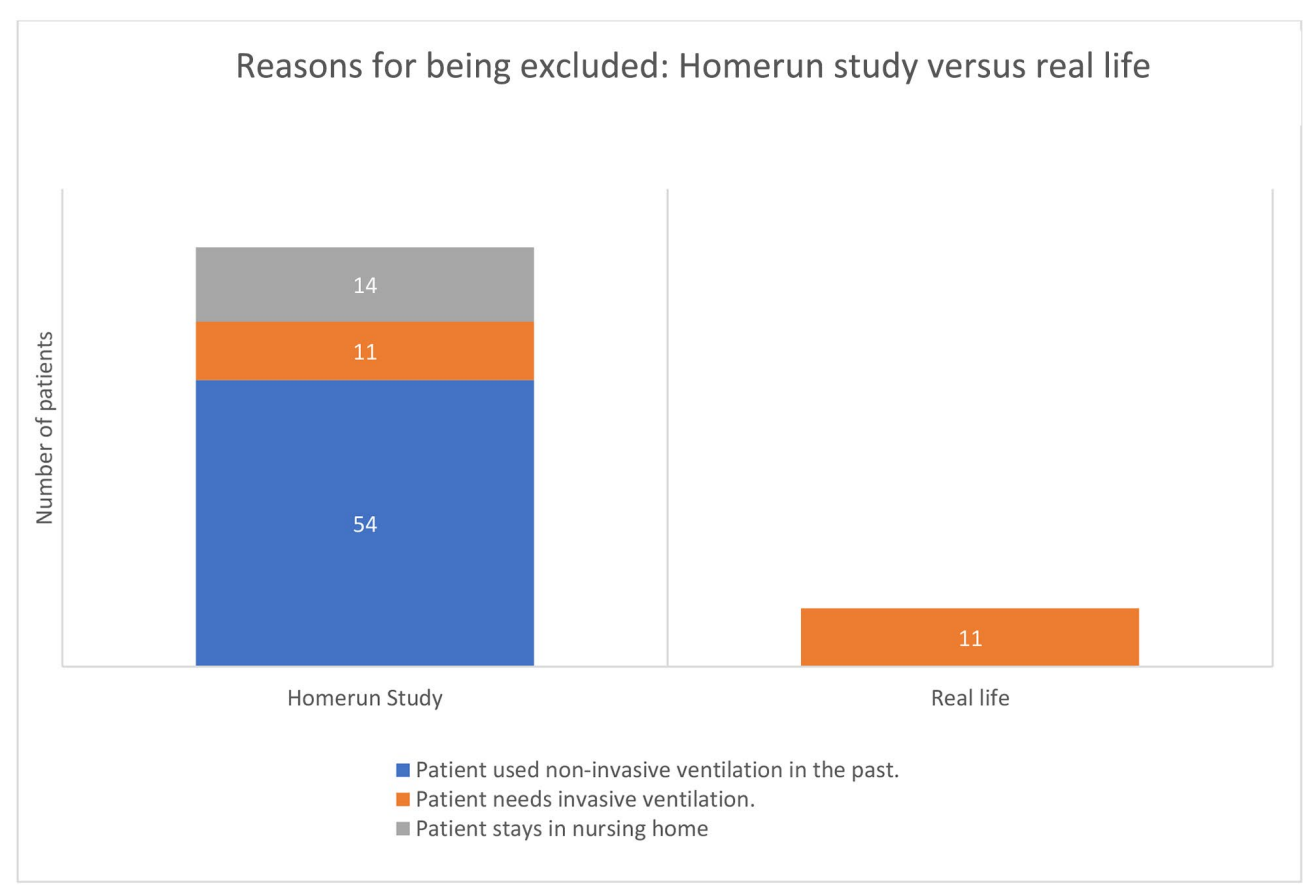
Distribution of patients fulfilling the inclusion criteria of the Homerun study versus real life

Two-hundred and forty-five participants, making up 48% of excluded participants, did not fulfil the inclusion criteria, primarily due to not having a NMD or TCA. However, other disorders characterised by hypoventilation might still be considered suitable candidates to start HMV at home in real life. Age limitation, an insufficient supportive network, and lack of motivation would remain reasons for not initiating HMV at home in real life (Fig. 2). Especially in ALS patient, although there is a normocapnia and absence of orthopnea there can be reasons, as experienced extensive work of breathing or frequent pulmonary infections, to start HMV in this exceptional cases.

### Patients fulfilling the exclusion criteria

**Fig. 3:**
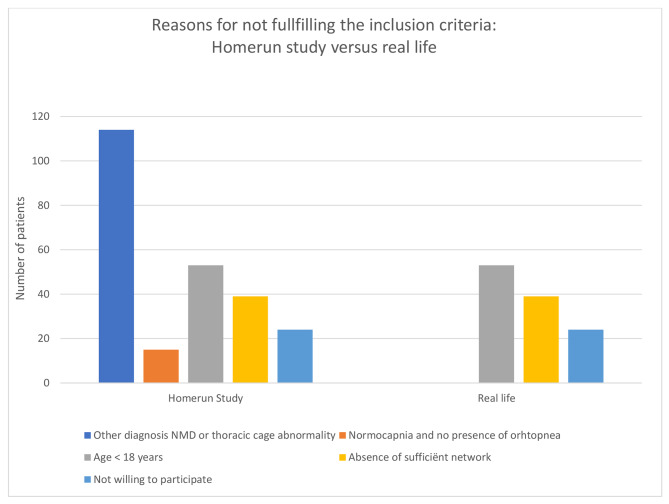
Distribution of patients meeting the exclusion criteria for the Homerun study versus real life

In the study 15% met the exclusion criteria, seventy-nine participants. From the 3 mentioned exclusion criteria, only the need for invasive ventilation renders starting HMV at home would be impossible in real life (Fig. 3).

### Comorbidity and organisational reasons for not participating

**Fig. 4:**
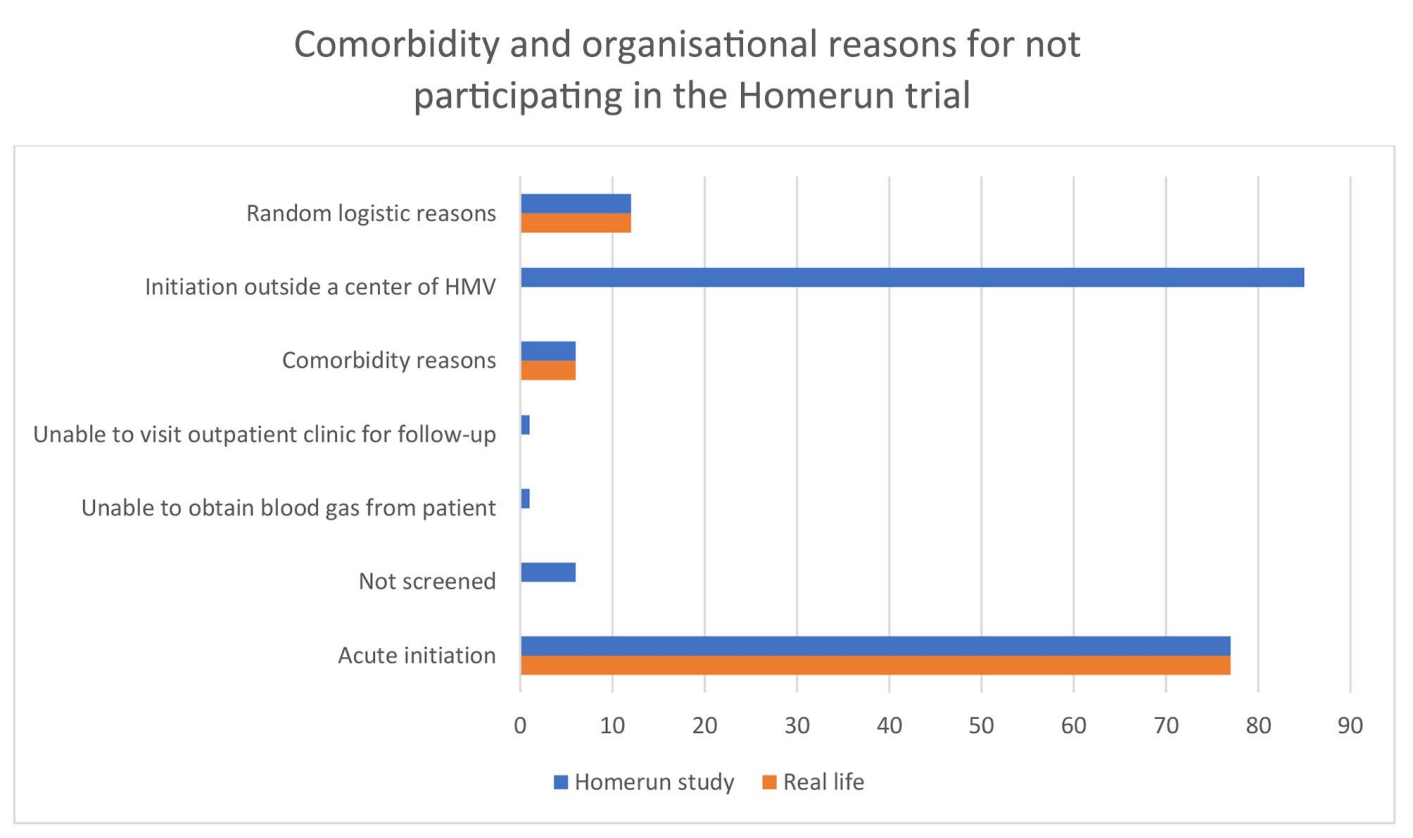
Comorbidity and organisational reasons for not participating in the Homerun study vs. real life

In this group a total of one hundred and eighty-eight patients had reasons for not participating. Due to logistical issues, 85 patients started HMV under guidance and supervision of an academic center of home mechanical ventilation in-hospital but outside one of the 4 HMV hospitals and could therefore not be randomized. Of note, if these patients met the inclusion criteria and would not have been excluded based on exclusion criteria, they could have initiated HMV at home. Unfortunately, inclusion in the Homerun trial was not discussed with these patients. The other reasons for non-participation related to comorbidity and organisational issues are presented in Fig. 4.

**Fig. 5:**
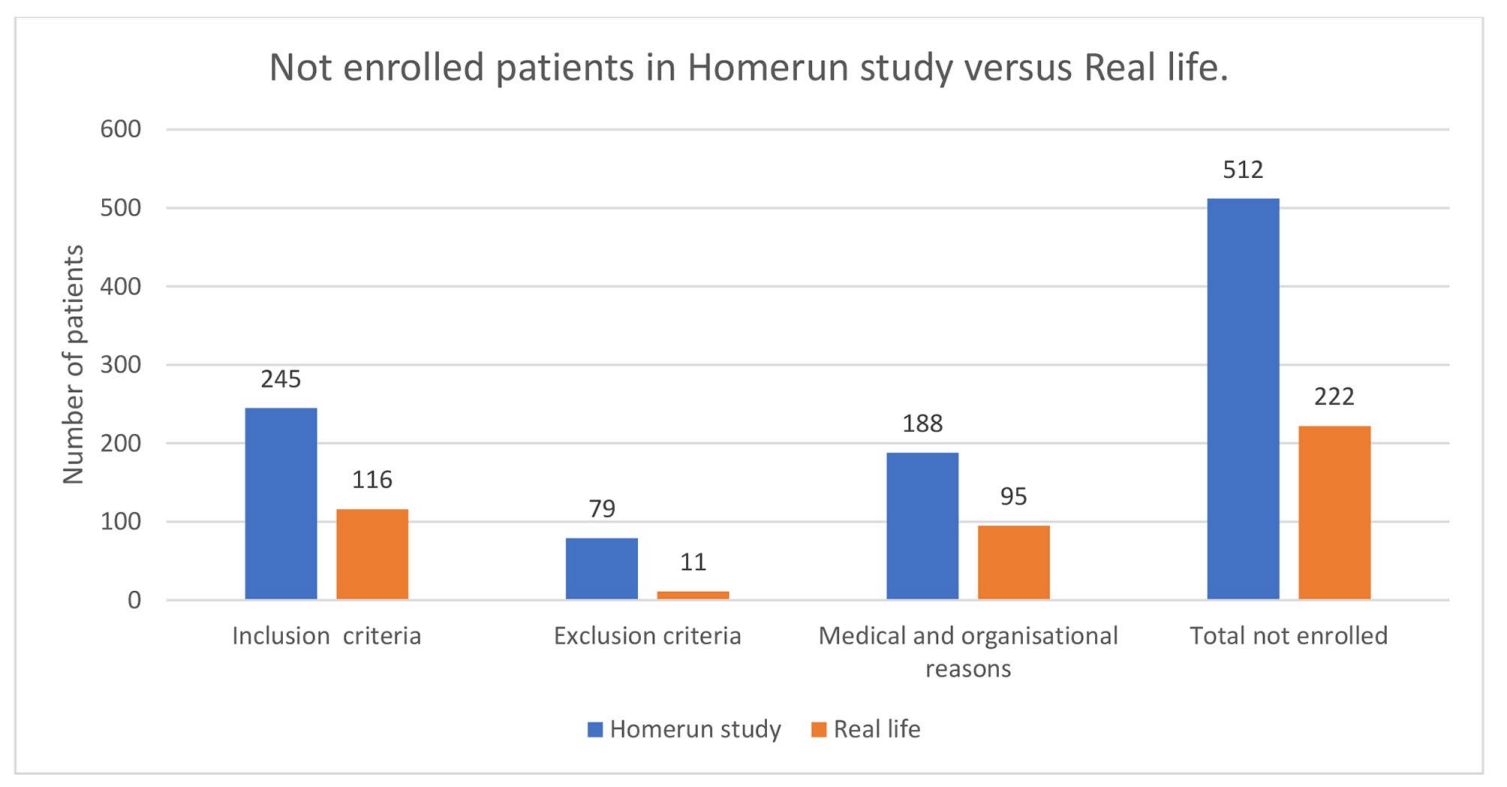
Summary of reasons for not starting HMV at home by category for the Homerun study vs. real life

In summary, while 512 patients were excluded from the Homerun study, in real life up to 290 of these could have started HMV at home, with the remaining 222 unsuitable of initiation of therapy at home due to various reasons (Fig. 5).

## Discussion

This study shows that initiation of HMV at home can be offered to larger group of patients than was initially thought based on in the Homerun study results. This is important as the HOMERUN study suggests that starting HMV at home is only feasible in a very select group of patients with an NMD or thoracic cage problem, this suggestion could be extended to the study of Hazenberg which used the same in- and exclusion criteria [2, 3]. Based on a recent study by Duiverman and colleagues, it has been shown chronic obstructive pulmonary disease (COPD) patients, which represent 13% of patients being referred for HMV, can be commenced on HMV at home as well [4]. Considering the other “Inclusion-related factors” mentioned in group 1, that might prohibit starting HMV at home, it remains difficult to upgrade an insufficient social network or improve patient motivation. While staying at home for starting therapy could positively influence the motivation for therapy, although we have no proof for this statement. Other actions to make the initiation at home more attractive with regards to motivation could be obtained by education and raising more awareness that starting HMV at home is safe and equally effective as compared to an inpatient start. To justify HMV initiation in patients under 18 years of age, similar studies as the Homerun trial need to be carried out in younger patients. Lastly, the exact reasons why patients declined to participate in the study were not obtained. Better communication of the positive results combined with sharing the experiences of other patients might take away the possible burden(s) anticipated by these patients.

With regard to the exclusion criteria, it is obvious that the start of tracheostomized ventilation and its complexity of care is only feasible in a high care unit. However, other criteria for exclusion from the Homerun study do not make initiation of HMV at home impossible in real life. a Starting HMV in a nursing home is also feasible as nurses are available 24/7 and care can be provided instantly.

The last category for not participating is more complex and diverse as shown in Fig. 4, but two reasons are relevant for discussion. During the Homerun trial, 85 patients started HMV in a regional hospital, under guidance and supervision of an academic center of home mechanical ventilation and inclusion in the Homerun study was not discussed as a possibility; it is highly conceivable that at least some of these patients would have qualified for HMV initiation at home. Regarding patients exclusion due to acute initiation of NIV, we know some patients may deteriorate acutely due to an infection, while other patients may gradually worsen over weeks or months. This latter group may have benefitted from home-initiated ventilation if identified and referred to a HMV centre at an earlier stage of their disease process. Education and improved awareness of health care professionals regarding positive effects of HMV in patients with or at risk of hypoventilation due to NMD or TD is important to reduce the number of patients needing start HMV in an acute setting.

Comparing this current study with previous work is difficult as we found only one study comparing hospital adaptation to HMV versus home adaptation [5]. While this observational prospective study showed improvements in both groups in terms of arterial blood gases and quality of life, however, it did not investigate reasons behind non-participation [5]. Several studies have investigated initiation of HMV in an outpatient setting compared to inpatient HMV initiation. Although reasons for not initiating HMV as an outpatient service were identified, it was unclear whether these reasons were primarily study-related or due to real-life circumstances. [6–9].

## Conclusion

This study suggests that up to 55% of patients who did not participate in the Homerun study could have started HMV at home in real life. Based on the expected growth of patients who need HMV in the future, we should reconsider our current clinical HMV initiation process and start HMV at home rather than at the hospital whenever a patient is considered eligible based on real-life criteria. Investments in the telemonitoring, proper training of staff, and organisational restructuring are needed to make this happen.

## Data Availability

The datasets used and analyzed during the current study are available from the corresponding author on reasonable request.

## References

[1] Cantero C, Adler D, Pasquina P (2020). Long-Term Noninvasive Ventilation in the Geneva Lake Area: Indications, Prevalence, and Modalities. Chest.

[2] van den Biggelaar RJM, Hazenberg A, Cobben NAM, Gaytant MA, Vermeulen KM, Wijkstra PJ. A Randomized Trial of Initiation of Chronic Noninvasive Mechanical Ventilation at Home vs In-Hospital in Patients With Neuromuscular Disease and Thoracic Cage Disorder: The Dutch Homerun Trial. Chest. 2020. 10.1016/j.chest.2020.07.007 32682770

[3] Hazenberg A, Kerstjens HA, Prins SC, Vermeulen KM, Wijkstra PJ (2014). Initiation of home mechanical ventilation at home: a randomised controlled trial of efficacy, feasibility and costs. Respir Med.

[4] Duiverman ML, Vonk JM, Bladder G, et al. Home initiation of chronic non-invasive ventilation in COPD patients with chronic hypercapnic respiratory failure: a randomised controlled trial. Thorax. 2019. 10.1136/thoraxjnl-2019-213303 PMC7063397 31484786

[5] Doménech-Clar R, Nauffal-Manssur D, Compte-Torrero L, Rosales-Almazán MD, Martínez-Pérez E, Soriano-Melchor E (2008). Adaptation and follow-up to noninvasive home mechanical ventilation: ambulatory versus hospital. Respir Med.

[6] Chatwin M, Nickol AH, Morrell MJ, Polkey MI, Simonds AK (2008). Randomised trial of inpatient versus outpatient initiation of home mechanical ventilation in patients with nocturnal hypoventilation. Respir Med.

[7] Pallero M, Puy C, Guell R (2014). Ambulatory adaptation to noninvasive ventilation in restrictive pulmonary disease: a randomized trial with cost assessment. Respir Med.

[8] Bertella E, Banfi P, Paneroni M (2017). Early initiation of night-time NIV in an outpatient setting: a randomized non-inferiority study in ALS patients. Eur J Phys Rehabil Med.

[9] Sheers N, Berlowitz DJ, Rautela L, Batchelder I, Hopkinson K, Howard ME (2014). Improved survival with an ambulatory model of non-invasive ventilation implementation in motor neuron disease. Amyotroph lateral Scler frontotemporal degeneration.

